# Online Dietary Intake Estimation: Reproducibility and Validity of the Food4Me Food Frequency Questionnaire Against a 4-Day Weighed Food Record

**DOI:** 10.2196/jmir.3355

**Published:** 2014-08-11

**Authors:** Rosalind Fallaize, Hannah Forster, Anna L Macready, Marianne C Walsh, John C Mathers, Lorraine Brennan, Eileen R Gibney, Michael J Gibney, Julie A Lovegrove

**Affiliations:** ^1^Hugh Sinclair Unit of Human Nutrition and Institute for Cardiovascular and Metabolic ResearchDepartment of Food and Nutritional SciencesUniversity of ReadingReadingUnited Kingdom; ^2^UCD Institute of Food and HealthUCD Centre for Molecular InnovationUniversity College DublinDublinIreland; ^3^Human Nutrition Research CentreInstitute for Health and AgeingNewcastle UniversityNewcastleUnited Kingdom

**Keywords:** food frequency questionnaire, weighed food record, validity, reproducibility, dietary assessment, Food4Me, Web-based

## Abstract

**Background:**

Advances in nutritional assessment are continuing to embrace developments in computer technology. The online Food4Me food frequency questionnaire (FFQ) was created as an electronic system for the collection of nutrient intake data. To ensure its accuracy in assessing both nutrient and food group intake, further validation against data obtained using a reliable, but independent, instrument and assessment of its reproducibility are required.

**Objective:**

The aim was to assess the reproducibility and validity of the Food4Me FFQ against a 4-day weighed food record (WFR).

**Methods:**

Reproducibility of the Food4Me FFQ was assessed using test-retest methodology by asking participants to complete the FFQ on 2 occasions 4 weeks apart. To assess the validity of the Food4Me FFQ against the 4-day WFR, half the participants were also asked to complete a 4-day WFR 1 week after the first administration of the Food4Me FFQ. Level of agreement between nutrient and food group intakes estimated by the repeated Food4Me FFQ and the Food4Me FFQ and 4-day WFR were evaluated using Bland-Altman methodology and classification into quartiles of daily intake. Crude unadjusted correlation coefficients were also calculated for nutrient and food group intakes.

**Results:**

In total, 100 people participated in the assessment of reproducibility (mean age 32, SD 12 years), and 49 of these (mean age 27, SD 8 years) also took part in the assessment of validity. Crude unadjusted correlations for repeated Food4Me FFQ ranged from .65 (vitamin D) to .90 (alcohol). The mean cross-classification into “exact agreement plus adjacent” was 92% for both nutrient and food group intakes, and Bland-Altman plots showed good agreement for energy-adjusted macronutrient intakes. Agreement between the Food4Me FFQ and 4-day WFR varied, with crude unadjusted correlations ranging from .23 (vitamin D) to .65 (protein, % total energy) for nutrient intakes and .11 (soups, sauces and miscellaneous foods) to .73 (yogurts) for food group intake. The mean cross-classification into “exact agreement plus adjacent” was 80% and 78% for nutrient and food group intake, respectively. There were no significant differences between energy intakes estimated using the Food4Me FFQ and 4-day WFR, and Bland-Altman plots showed good agreement for both energy and energy-controlled nutrient intakes.

**Conclusions:**

The results demonstrate that the online Food4Me FFQ is reproducible for assessing nutrient and food group intake and has moderate agreement with the 4-day WFR for assessing energy and energy-adjusted nutrient intakes. The Food4Me FFQ is a suitable online tool for assessing dietary intake in healthy adults.

## Introduction

Given the continuing rise in some noncommunicable diseases and the growing burden of diet-related ill health [1-3], researchers are seeking new and innovative ways of facilitating dietary change. These include the application of digital technologies, which are revolutionizing the delivery of health-related services because of their reduced costs and wide reach. Online interventions are particularly promising because they have the potential to increase exposure to health promotion material. Recent estimates show that Internet use has increased by >150% in North America and by nearly 400% in Europe since 2000, with a total of 78.6% and 63.2% of these populations, respectively, now classified as Internet users [4]. Given their lower costs, Internet-based services have the potential to enhance the cost-benefit ratio for interventions aimed at prevention of diet-related noncommunicable diseases [5-6]. Furthermore, interactive Web-based interventions have been shown to increase patient activation and self-management capabilities in chronically ill adults [7] and enhance weight loss in obese individuals (compared with non-Web-based interventions) [8].

To quantify dietary change in response to an intervention, an accurate and validated means of assessing food intake is essential [9]. Population-level food intake is usually assessed in 1 of 3 ways: a food frequency questionnaire (FFQ), 24-hour recall, or estimated or weighed food record (WFR). The WFR, which involves weighing all foods and drinks consumed over a 3-7 day period, is often considered the most accurate measure of intake and has been referred to as the imperfect gold standard [10]. However, prospective recording of food consumption can alter the type and quantity of foods eaten and, therefore, introduce bias into the estimate of food intake [11-13]. The FFQ and 24-hour recall, which rely on retrospective recording of food consumption, are also prone to reporting bias, including overestimated consumption of “healthy” foods, such as fruit and vegetables, and underestimation of “unhealthy” food intake. WFR require participants to be highly motivated and are labor-intensive for both participants and researchers. Conversely, FFQ are inexpensive to process and can be self-administered electronically, making them suitable for online interventions. Other advantages include reducing paper use, postage costs, and the space; security; and organization required for paper file storage [14]. For this reason, FFQ are most commonly used in large-scale epidemiological and intervention studies to determine food and nutrient intake [15].

The present research was conducted as part of the Food4Me study, which aims to test the utility of online personalized dietary advice using an online FFQ to assess dietary intake [16,17]. The Food4Me FFQ includes 157 food items and food portion photographs and has been described previously by Forster et al [18]. FFQ are generally validated against existing dietary assessment methods, such as WFR [19], and several FFQ have been validated for electronic and online use recently [14,20-22].

The Food4Me FFQ has been shown to have good agreement with the European Prospective Investigation of Cancer (EPIC)-Norfolk FFQ for the estimation of energy-adjusted nutrient intakes [18]. The aim of this study is to further validate the Food4Me FFQ against a WFR and to assess its reproducibility using a test-retest methodology.

## Methods

### Study Sample

To accurately estimate the Bland-Altman limits of agreement between 2 methods, a sample size of 50-100 is required [23]. Allowing for 20% dropout, 121 participants aged ≥18 years were recruited from the University of Reading, UK, via email and poster advertising. Participants were provided with a study information sheet before participation and were asked to sign an informed consent form. A participant information form, which included self-reported weight and height measurements, was used to assess suitability for the study. Individuals reporting health issues or ill health, self-reported or diagnosed food intolerances, or special nutritional requirements (eg, pregnancy or lactation) were ineligible to participate. Ethical approval for the study was obtained from the School of Chemistry, Food and Pharmacy Research Ethics Committee, University of Reading, UK (01-12-Lovegrove).

### Study Design

Reproducibility of the Food4Me FFQ was determined by asking participants to complete the questionnaire on 2 occasions 4 weeks apart, mimicking its application in the Food4Me study. To assess the validity of the FFQ against a 4-day WFR, half the sample (those recruited first) were asked to complete a 4-day WFR 1 week following the first administration of the Food4Me-FFQ. Participants who completed both the Food4Me FFQ and 4-day WFR were also asked to complete a dietary record usability-rating questionnaire on Survey Monkey (Survey Monkey Inc, Palo Alto, CA, USA) in the week following the completion of the second Food4Me FFQ. The usability-rating questionnaire included questions about ease of use and willingness to complete the records. Participants were asked not to change their diet during the study.

### Weighed Food Record

Participants were asked to record all foods and beverages consumed over a nonconsecutive 4-day period that included 3 weekdays (Monday to Thursday) and 1 weekend day (Saturday to Sunday). Before completing the WFR, participants were coached on how to describe food products by a dietitian and provided with weighing scales (Salter Disc Electronic Kitchen Scales SKU# 1036 WHSSDR). When participants were unable to provide weighed portion size information, this was estimated retrospectively within 1 week using the Ministry of Agriculture, Fisheries and Food Portion Size Atlas [24].

### Food4Me FFQ

**Figure 1:**
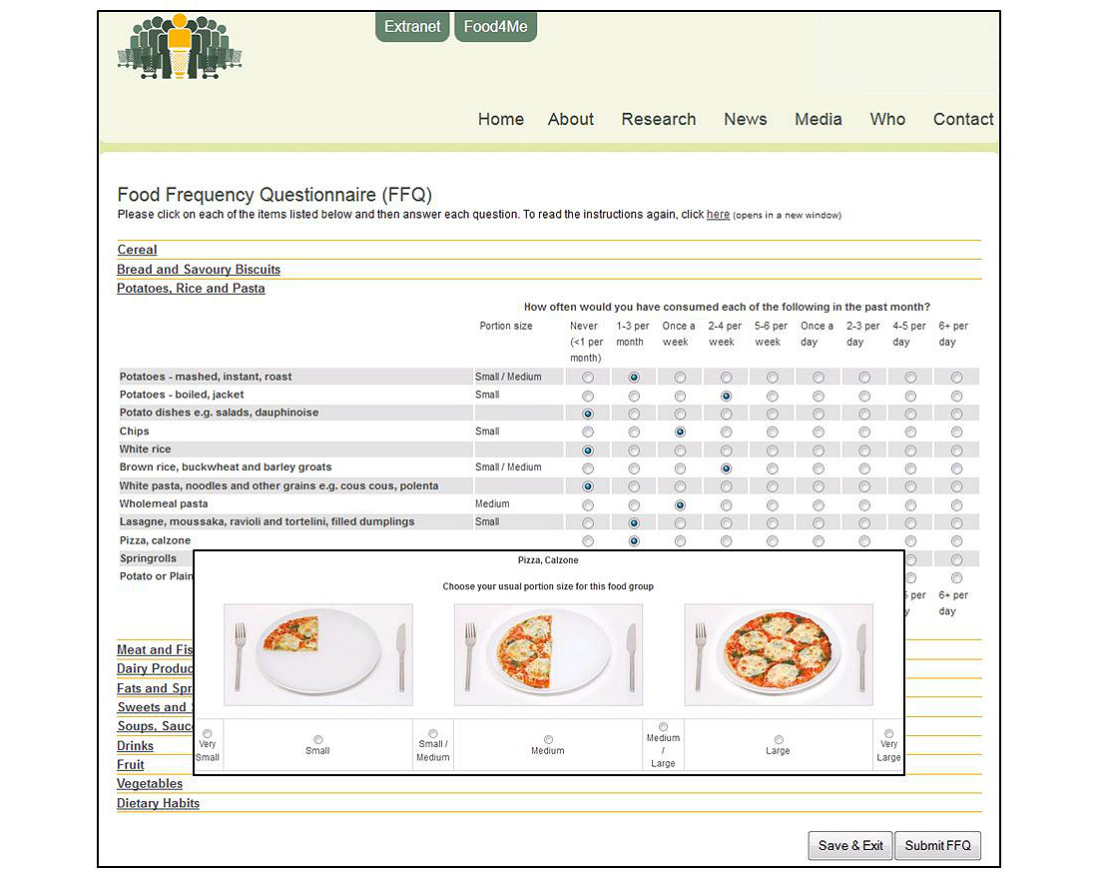
Screenshot of the online Food4Me food frequency questionnaire.

The self-administered Food4Me FFQ is an online, semiquantitative food frequency questionnaire (developed by University College Dublin and Crème Software Ltd). To complete the questionnaire, participants were provided with a website address and unique log-in details. On logging into the server, participants were directed to a webpage containing detailed instructions on how to complete the FFQ. The questionnaire contained questions on the average consumption of 157 food items over the previous month. The food items were divided into the following 11 categories: (1) cereal, (2) bread and savory biscuits, (3) potatoes, rice and pasta, (4) meat and fish, (5) dairy products and fat, (6) fats and spreads, (7) sweets and snacks, (8) soups, sauces and spreads, (9) drinks, (10) fruit, and (11) vegetables. During completion of the Food4Me FFQ, participants were required to provide information on frequency of consumption and portion size. Frequency of consumption was measured by selecting one of the following options: never or less than once a month, 1-3 times a month, once a week, 2-4 times a week, 5-6 times per week, once a day, 2-3 times per day, 5-6 times per day, and >6 times per day. Food portion size was estimated using photographs. Each food item had 3 photographs representing small, medium, and large portions and these descriptors were provided below the appropriate image. Participants could select one of the following options: very small, small, small/medium, medium, medium/large, large, or very large which were linked electronically to portion sizes (in grams) (see Figure 1). Food intake (g/day) was calculated by multiplying frequency of consumption by the specified portion size (see Forster et al for detailed methods [18]). Further screenshots of the online Food4Me FFQ are shown in Multimedia Appendix 1.

### Dietary Record Usability-Rating Questionnaire

The dietary record usability-rating questionnaire was comprised of 5 questions about the completion of the Food4Me FFQ and the 4-day WFR. Participants who completed both the Food4Me FFQ and the 4-day WFR (n=49) were asked to select one of the following responses to indicate their level of agreement: strongly agree, agree, neither agree nor disagree, disagree, or strongly disagree to the following questions:

1. I found the Food4Me FFQ / 4-day WFR easy to complete
2. I found the Food4Me FFQ / 4-day WFR too time consuming
3. I found the Food4Me FFQ / 4-day WFR interesting to complete
4. I found the Food4Me FFQ / 4-day WFR made me reflect on my intake
5. In the future I would be willing to complete more Food4Me FFQ/ 4-day WFR

### Misreporting

The Henry equation [25] was used to calculate basal metabolic rate (BMR), and BMR was multiplied by a physical activity level (PAL) of 1.1 to calculate the lowest possible estimated energy requirements (EER) for each participant [26]. Participants reporting energy intakes lower than their EER were classified as underreporters. Participants reporting a daily energy intake greater than 4500 kcal, which is considered implausibly high, were excluded from the analysis [27].

### Nutritional Intake Analysis

Estimated nutritional intake data from the Food4Me FFQ were generated automatically by the online Food4Me programmed system, as described by Forster et al [18]. Composition of the food items listed in the FFQ were derived from WISP (Tinuviel Software, Anglesey, UK) [28] and modified to include recipes of composite dishes, generic commercial foods, new foods on the market, and current manufacturers information. The 4-day WFR intakes were analyzed using WISP (Tinuviel Software, Anglesey, UK) [28]. For the purpose of the current study, consumption of dietary supplements was not included in the analyses.

### Statistical Analysis

Statistical analyses were performed using SPSS version 20.0 (IBM Corp, Armonk, NY, USA). Mean nutrient intakes and standard deviations were calculated for baseline characteristics, repeated Food4Me FFQ, and 4-day WFR. Differences in participant characteristics and energy intakes (kcal) were assessed using a paired 2-sample *t* test. Nutrient intakes were compared using general linear model (GLM) analysis controlling for energy and gender where there was significant interaction between gender and nutrient intake. Data were checked for normality using the Shapiro-Wilk test and, depending on the outcome, the association between dietary intake methods and repeated Food4Me FFQ were assessed using either Pearson product-moment correlation or Spearman correlation coefficient (SCC, rho). A *P* value of <.05 was considered statistically significant.

The relative agreement between the dietary intake methods and repeated Food4Me FFQ was assessed using cross-classification of nutrient intakes to estimate the percentage of participants classified into quartiles as follows: exact agreement (percentage of cases classified into the same quartile), exact agreement plus adjacent (percentage of cases cross-classified into the same or adjacent quartile), disagreement (percentage of cases cross-classified 2 quartiles apart), and extreme disagreement (percentage of cases cross-classified into extreme quartiles). For intakes of energy and macronutrients, the Bland-Altman method [29] was used to further assess the limits of agreement between the 2 methods (Food4Me FFQ and WFR) and between the repeated Food4Me FFQ. As per the Bland-Altman methodology, dietary records were considered comparable/ repeatable if greater than 95% of data plots lay within 2 standard deviations of the mean. GraphPad PRISM version 6 was used to produce the Bland-Altman plots (GraphPad Software, Inc, La Jolla, CA, USA).

Differences in food group intakes between the FFQ and WFR and repeated Food4Me FFQ were also examined. For this purpose, food items in the Food4Me FFQ and 4-day WFR were arranged into 35 food groups as per previous validation by Forster et al [18]. SCC were calculated to assess the strength of association between methods for estimated intakes of the 35 food groups. To assess the relative agreement between the dietary methods and repeated FFQ for daily food group intake, food groups were also cross-classified to estimate the percentage of participants classified by the 2 methods into quartiles of exact agreement, exact agreement plus adjacent, disagreement, and extreme disagreement.

## Results

### Summary

**Figure 2:**
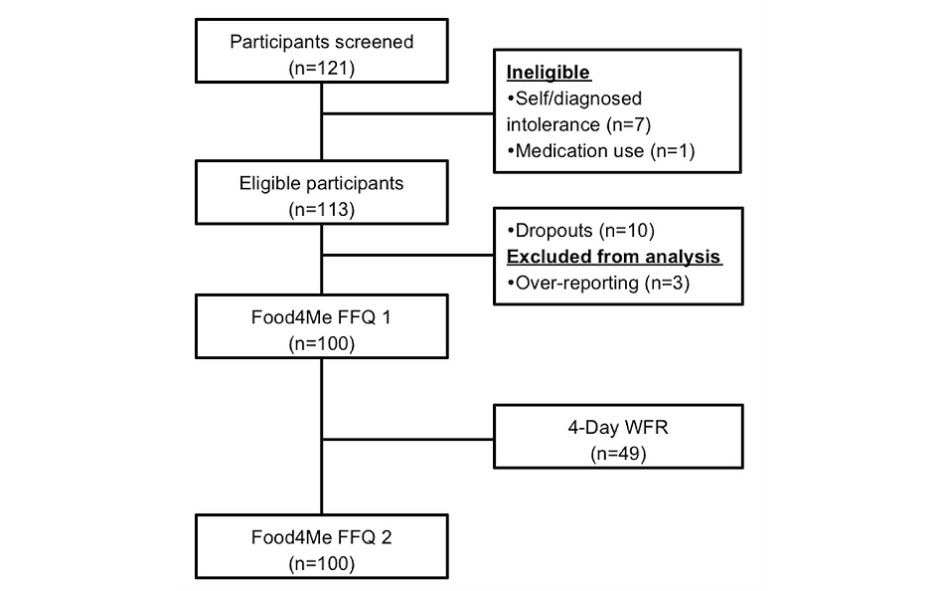
Flow of participants through the study.

A total of 121 participants were screened for inclusion in the study, of which 113 were deemed eligible. Reasons for exclusion included self-diagnosed food intolerance (n=7) and medication use (n=1). Before completion, 10 participants dropped out of the study and a further 3 were excluded from analysis due to a reported energy intake >4500 kcal [26]. The final dataset for analysis included 100 participants, of whom 49 had also completed the 4-day WFR, as illustrated in Figure 2. A total of 48 participants completed the Diet Record Usability-Rating Questionnaire.

### Overview of the Study Population

Self-reported demographic characteristics of the participants in the reproducibility and validation study are shown in Table 1. No significant differences were observed between age and body mass index (BMI) for males and females. Participants who completed the WFR (validation study) were, on average, 4.6 years younger than the participant group as a whole.

**Table 1:** Demographic characteristics of the participants who completed the validation and reproducibility studies according to gender.

Study		N	Demographic characteristics, mean (SD)	
			Age (years)	BMI (kg/m^2^)^a^
**Reproducibility**				
	Males	31	30.1 (12.7)	24.3 (3.1)
	Females	69	32.1 (11.6)	23.3 (3.3)
	All	100	31.5 (11.9)	23.6 (3.3)
**Validation**				
	Males	15	24.2 (7.6)	23.1 (3.2)
	Females	34	27.9 (8.6)	22.2 (2.6)
	All	49	26.9 (8.4)	22.5 (2.8)

^a^BMI based on self-reported weight and height.

### Reproducibility of the Food4Me FFQ

#### Comparison of Nutrient Intakes Between Repeated Food4Me FFQ

Mean energy and nutrient intakes estimated by repeated measures of the Food4Me FFQ (FFQ1 and FFQ2) are presented in Table 2. Estimated energy intakes were significantly higher in the first administration of the FFQ compared with the second administration (difference=135 kcal/day, equivalent to 6.5% higher, *P*<.05). With the exception of carbohydrate, no significant differences were observed between macronutrient and micronutrient intakes estimated by FFQ1 and FFQ2. Overall, the Food4Me FFQ showed good reproducibility for energy-adjusted nutrient intakes. A total of 16 participants were found to underreport in both FFQ with a further 3 underreporting in FFQ1 and 5 in FFQ2. The removal of underreporters from both FFQ (n=24) did not impact on the reproducibility of the questionnaire (data not shown).

**Figure 3:**
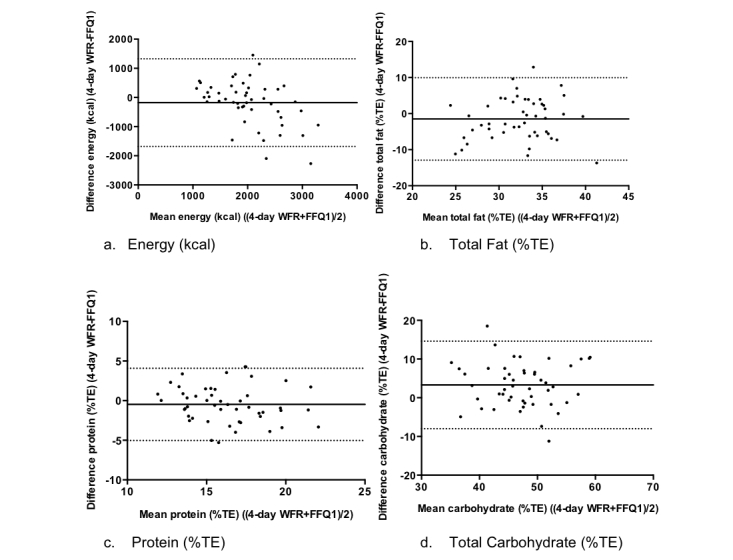
Reproducibility study Bland-Altman plots for (a) energy, (b) total fat, (c) protein, and (d) carbohydrate with the mean difference and limits of agreement. The solid line represents the mean difference and the dotted lines represent the limits of agreement.

Bland-Altman plots for estimates of energy (kcal), total fat (% total energy, TE), protein (%TE) and carbohydrate (%TE) intakes are shown in Figure 3. The Food4Me FFQ showed good reproducibility for the estimation of daily protein intake, with less than 5% of cases falling outside of the limits of agreement. For energy and total fat intake, 6% of cases fell outside of the limits of agreement and for carbohydrate 7%, indicating similar reproducibility. The mean difference (bias) between energy intakes was relatively small (135 kcal/day) with greater values being estimated at FFQ1. Conversely, estimates of energy-adjusted protein and total fat intake were higher at FFQ2 with biases of –0.22 %TE and –1.23 %TE, respectively. In contrast with the energy-adjusted macronutrient intakes, variation between estimates of energy increased with higher mean energy intakes (Figure 3), suggesting poorer reproducibility for those participants reporting higher energy intakes.

Correlation coefficients for estimates of energy and nutrient intakes between repeated administrations of the Food4Me FFQ are shown in Table 3. Correlation coefficients ranged from .65 (vitamin D) to .90 (alcohol) with a mean value of .75. Correlations were significant for all nutrients (*P*<.01). The cross-classification of quartiles of mean estimated daily energy and nutrient intakes between repeated administrations of the Food4Me FFQ is also shown in Table 3. The percentage of participants classified into quartiles of exact agreement ranged from 45% (polyunsaturated fatty acids %TE) to 74% (vitamin A retinol equivalents, RE). For classifications of exact agreement plus adjacent, values were consistently high, ranging from 87% (vitamin D) to 98% (vitamin A RE). The mean percentage of participants classified into quartiles of disagreement was 7% with less than 1% of participants on average classified into extreme disagreement.

**Table 2:** Mean daily energy and nutrient intakes estimated by repeated measures of the online Food4Me FFQ and general linear model (GLM) results (N=100).

Nutrient^a^	Questionnaire, mean (SD)		GLM analysis, *P*	
	Food4Me FFQ1	Food4Me FFQ2	Controlled for energy	Controlled for energy and gender^b^
Energy (kcal)	2223.8 (766.2)	2088.8 (705.4)	.008^c^	—
Total fat (g)	85.3 (33.8)	82.0 (32.5)	.24	.24
Total fat (%TE)	34.1 (4.9)	35.1 (6.1)	.21	.21
SFA (g)	33.2 (14.2)	32.2 (14.5)	.13	.13
SFA (%TE)	13.2 (2.4)	13.6 (3.0)	.23	.23
MUFA (g)	32.0 (13.4)	30.6 (12.7)	.38	.38
MUFA (%TE)	12.8 (2.5)	13.1 (2.8)	.36	.36
PUFA (g)	14.4 (5.6)	13.7 (5.3)	.91	.98
PUFA (%TE)	5.8 (1.27)	6.0 (1.5)	.48	.62
Omega 3 (g)	1.70 (0.71)	1.61 (0.66)	.92	.92
Protein (g)	90.6 (35.2)	84.4 (30.8)	.70	.70
Protein (%TE)	16.3 (2.8)	16.4 (4.5)	.84	.84
Carbohydrate (g)	263.9 (87.6)	238.6 (89.6)	.06	.03
Carbohydrate (%TE)	45.1 (6.5)	43.1 (7.9)	.05	.03
Total sugars (g)	125.6 (49.3)	115.9 (56.1)	.65	.79
Total sugars (%TE)	21.4 (5.8)	20.6 (5.8)	.31	.32
Alcohol (g)	12.9 (16.2)	12.8 (15.7)	.61	.60
Calcium (mg)	1085.0 (378.1)	1008.0 (416.5)	.59	.64
Total folate (µg)	361.0 (120.0)	335.9 (416.5)	.43	.43
Iron (mg)	14.9 (5.3)	13.4 (4.3)	.05	.05
Total carotene (µg)	6209.6 (4590.8)	5482.1 (3645.2)	.43	.60
Riboflavin (mg)	2.24 (0.80)	2.11 (0.89)	.87	.98
Thiamin (mg)	2.87 (2.80)	2.91 (3.33)	.76	.76
Vitamin B6 (mg)	2.54 (0.86)	2.35 (0.78)	.31	.31
Vitamin B12 (µg)	7.32 (3.57)	6.72 (3.60)	.64	.64
Vitamin C (mg)	167.8 (82.5)	153.8 (74.7)	.42	.51
Vitamin A RE (µg)	1160.9 (1015.8)	1057.8 (907.9)	.79	.79
Retinol (µg)	502.9 (408.0)	470.1 (400.5)	.99	.99
Vitamin D (µg)	3.89 (2.39)	3.51 (1.90)	.48	.38
Vitamin E (mg)	10.61 (4.05)	9.77 (3.86)	.39	.29
Salt (g)	6.30 (2.70)	5.92 (2.24)	.97	.97

^a^MUFA: monounsaturated fatty acids; PUFA: polyunsaturated fatty acids; RE: retinol equivalents; SFA: saturated fatty acids; TE: total energy.

^b^Controlled for gender where appropriate. No significant interactions were observed between method and gender.

^c^ *P* value derived using 2-samples paired *t* test.

**Table 3:** Unadjusted correlation coefficients and cross-classification of quartiles of mean energy and nutrient intakes derived from repeat measures of the online Food4Me FFQ (N=100).

Nutrient^a^	Correlation^b^	Quartiles, %			
		Exact agreement^c^	Exact agreement plus adjacent^d^	Disagreement^e^	Extreme disagreement^f^
Energy (kcal)	.77^g^	57	90	9	1
Total fat (g)	.81	64	92	8	0
Total fat (%TE)	.72	56	91	7	2
SFA (g)	.81	60	91	9	0
SFA (%TE)	.70	46	88	12	0
MUFA (g)	.80	51	96	4	0
MUFA (%TE)	.70	53	89	10	1
PUFA (g)	.78	51	95	3	2
PUFA (%TE)	.68	45	92	6	2
Omega 3 (g)	.78	58	91	9	0
Protein (g)	.80	56	88	12	0
Protein (%TE)	.73	59	93	7	0
Carbohydrate (g)	.74	53	96	4	0
Carbohydrate (%TE)	.73	62	89	9	2
Total sugars (g)	.77	66	94	4	2
Total sugars (%TE)	.69	61	88	11	1
Alcohol (g)	.90	70	96	4	0
Calcium (mg)	.73	55	92	7	1
Total folate (µg)	.74	53	93	6	1
Iron (mg)	.75	53	95	4	1
Total carotene (µg)	.76	60	90	10	0
Riboflavin (mg)	.73	56	90	8	2
Thiamin (mg)	.71	51	91	6	3
Vitamin B6 (mg)	.72	56	89	10	1
Vitamin B12 (µg)	.73	64	95	5	0
Vitamin C (mg)	.72	60	95	5	0
Vitamin A (RE) (µg)	.90	74	98	2	0
Retinol (µg)	.67	50	90	7	3
Vitamin D (µg)	.65	52	87	12	1
Vitamin E (mg)	.75	56	91	7	2
Salt (g)	.78	57	90	8	2

^a^MUFA: monounsaturated fatty acids; PUFA: polyunsaturated fatty acids; RE: retinol equivalents; SFA: saturated fatty acids; TE: total energy.

^b^Correlation is significant at the .01 level (2-tailed) for all nutrients analyzed.

^c^Exact agreement, % of cases cross-classified into the same quartile.

^d^Exact agreement plus adjacent, % of cases cross-classified into the same or adjacent quartile.

^e^Disagreement, % of cases cross-classified 2 quartiles apart.

^f^Extreme disagreement, % of cases cross-classified into extreme quartiles.

^g^Pearson correlation.

#### Comparison of Food Group Intakes Between Repeated Food4Me FFQ

To assess differences in food group intake between repeated administrations of the online Food4Me FFQ, food items were categorized into 35 food groups. Correlation coefficients and cross-classification of mean food group intakes are presented in Table 4. SCC ranged from .55 (tinned fruit or vegetables) to .92 (alcoholic beverages) with a mean value of .75. Correlations were significant for all food groups (*P*<.01). The percentage of participants classified into quartiles of exact agreement ranged from 46% (fats and oils) to 86% (tinned fruit or vegetables). For classifications of exact agreement plus adjacent values were consistently high, ranging from 81% (eggs and egg dishes) to 99% (alcoholic beverages). The mean percentage of participants classified into quartiles of disagreement was 7% and for extreme disagreement was 1%.

**Table 4:** Spearman correlation coefficients (SCC) and cross-classification of quartiles of food group intake derived from repeat measures of the online Food4Me FFQ (n=100).

Food group	SCC^a^	Quartile, %			
		Exact agreement^b^	Exact agreement plus adjacent^c^	Disagreement^d^	Extreme disagreement^e^
Rice, pasta, grains and starches	.78	56	92	8	0
Savories (lasagne, pizza)	.70	52	89	9	2
White bread (rolls, tortillas, crackers)	.83	62	94	6	0
Wholemeal, brown breads, and rolls	.77	60	91	8	1
Breakfast cereals and porridge	.90	67	96	4	0
Biscuits	.56	48	86	11	3
Cakes, pastries and buns	.64	52	87	10	3
Milk	.74	49	91	7	2
Cheeses	.66	52	87	11	2
Yogurts	.79	58	97	2	1
Ice cream, creams and desserts	.77	55	94	4	2
Eggs and egg dishes	.69	56	81	16	3
Fats and oils (eg, butter, low-fat spreads, hard cooking fats)	.64	46	89	8	3
Potatoes and potato dishes	.61	51	86	9	5
Chipped, fried & roasted potatoes	.61	57	87	12	1
Peas, beans and lentils and vegetable and pulse dishes	.75	62	92	7	1
Green vegetables	.76	54	93	6	1
Carrots	.68	54	90	8	2
Salad vegetables (eg, lettuce)	.77	58	92	6	2
Other vegetables (eg, onions)	.85	56	97	2	1
Tinned fruit or vegetables	.55	86	86	14	0
Bananas	.81	60	95	5	0
Other fruits (eg, apples, pears, oranges)	.86	61	97	3	0
Nuts and seeds, herbs and spices	.77	68	84	13	3
Fish and fish products/dishes	.84	57	95	5	0
Bacon and ham	.88	73	97	3	0
Red meat (eg, beef, veal, lamb, pork)	.74	67	90	9	1
Poultry (chicken and turkey)	.75	54	93	6	1
Meat products (eg, burgers, sausages, pies, processed meats)	.85	62	93	7	0
Alcoholic beverages	.92	71	99	1	0
Sugars, syrups, preserves, and sweeteners	.78	80	94	6	0
Confectionary and savory snacks	.73	52	92	8	0
Soups, sauces, and miscellaneous foods	.69	59	90	8	2
Teas and coffees	.85	69	96	3	1
Other beverages (eg, fruit juices, carbonated beverages, squash)	.75	54	95	4	1

^a^Correlation is significant at the .01 level (2-tailed) for all nutrients analyzed.

^b^Exact agreement, % of case cross-classified into the same quartile.

^c^Exact agreement plus adjacent, % of cases cross-classified into the same or adjacent quartile.

^d^Disagreement, % of cases cross-classified 2 quartiles apart.

^e^Extreme disagreement, % of cases cross-classified into extreme quartiles.

### Validation of the Food4Me FFQ

#### Comparison of Nutrient Intakes Between the Food4Me FFQ and 4-Day WFR

Mean energy and nutrient intakes estimated by the 4-day WFR and Food4Me FFQ (FFQ1) are presented in Table 5. There were no significant differences between estimates of energy intake. However, when underreporters had been removed (n=19), a significant difference in intake was observed (*P*<.05) (Table 5). In total, 12 participants underreported in the 4-day WFR and 15 underreported in the Food4Me FFQ1, with 8 of these underreporting in both methods.

**Table 5:** Mean daily energy and nutrient intakes estimated by online Food4Me FFQ and 4-day WFR and general linear model (GLM) results (n=49).

Nutrient^a^	Questionnaire, mean (SD)		GLM analysis, *P*	
	Food4Me FFQ1	4-day WFR	Controlled for energy	Controlled for energy and gender^b^
Energy (kcal)	2115.2 (809.1)	1936.9 (505.8)	.11^c^	—
Total fat (g)	79.6 (36.2)	68.6 (22.2)	.10	.10
Total fat (%TE)	33.1 (4.5)	31.6 (5.1)	.13	.13
SFA (g)	45.6 (15.6)	24.3 (10.4)	<.001	<.001
SFA (%TE)	13.1 (2.3)	11.0 (2.9)	<.001	<.001
MUFA (g)	29.8 (4.5)	21.4 (7.3)	<.001	<.001
MUFA (%TE)	12.4 (2.6)	9.8 (2.0)	<.001	<.001
PUFA (g)	12.7 (4.9)	10.7 (4.6)	.12	.12
PUFA (%TE)	5.44 (0.9)	4.97 (1.6)	.10	.10
Protein (g)	87.2 (36.0)	77.2 (21.4)	.31	.31
Protein (%TE)	16.5 (2.9)	16.1 (2.6)	.40	.40
Carbohydrate (g)	253.4 (94.1)	248.3 (54.9)	.20	.20
Carbohydrate (%TE)	45.6 (6.6)	48.9 (6.5)	.01	.01
Total sugars (g)	119.1 (46.7)	102.8 (37.8)	.18	.18
Total sugars (%TE)	21.5 (5.5)	20.1 (6.2)	.25	.25
Alcohol (g)	13.0 (14.5)	11.6 (22.2)	.50	.50
Calcium (mg)	1043.8 (386.8)	865.8 (285.5)	.003	.001
Total folate (µg)	337.6 (124.6)	273.8 (139.5)	.05	.11
Iron (mg)	14.1 (5.4)	13.0 (5.6)	.98	.98
Total carotene (µg)	5011.4 (3321.2)	2725.3 (2995.3)	.001	.001
Riboflavin (mg)	2.27 (0.83)	1.85 (0.82)	.04	.04
Thiamin (mg)	2.22 (1.56)	2.19 (3.26)	.98	.98
Vitamin B6 (mg)	2.44 (0.83)	2.09 (0.70)	.06	.06
Vitamin B12 (µg)	6.85 (3.31)	4.63 (2.16)	<.001	<.001
Vitamin C (mg)	148.2 (77.0)	106.6 (73.1)	.02	.05
Retinol (µg)	426.1 (330.3)	236.2 (137.7)	.001	.001
Vitamin D (µg)	3.47 (2.15)	2.55 (1.61)	.049	.049
Vitamin E (mg)	9.11 (3.36)	7.84 (2.77)	.13	.13
Salt (g)	5.91 (2.7)	6.48 (2.1)	<.001	<.001

^a^MUFA: monounsaturated fatty acids; PUFA: polyunsaturated fatty acids; RE: retinol equivalents; SFA: saturated fatty acids; TE: total energy.

^b^Controlled for gender where appropriate. No significant interactions were observed between method and gender.

^c^ *P* value derived using 2-samples paired *t* test.

After controlling for energy, estimated intakes of macronutrients were similar for both the WFR and the Food4Me FFQ with no significant differences between intakes of total fat (g, TE), polyunsaturated fatty acids (PUFA g, %TE), protein (g, %TE), carbohydrate (g), and total sugars (g, %TE) (Table 5). However, estimated intakes of saturated fatty acids (SFA) (g, %TE) and monounsaturated fatty acids (MUFA) (g, %TE) were significantly higher (*P*<.001), and estimated intake of carbohydrate (%TE) was significantly lower (*P*=.01), for the FFQ than for the WFR. For micronutrients, no significant differences were observed between energy-controlled estimates of folate, iron, thiamin, vitamin B6, and vitamin E. Estimated intakes of calcium, total carotene, riboflavin, vitamin B12, vitamin C, retinol, and vitamin D intakes were significantly different between 4-day WFR and FFQ1 (all were higher for the FFQ). After controlling for energy and, where appropriate, gender, vitamin C intakes were no longer significantly different. Removing underreporters from the dataset reduced the agreement between the 2 methods for folate, vitamin B6, and vitamin E, but improved agreement for energy-controlled carbohydrate and vitamin D, with no significant differences observed between estimates of these nutrients (data not shown).

**Figure 4:**
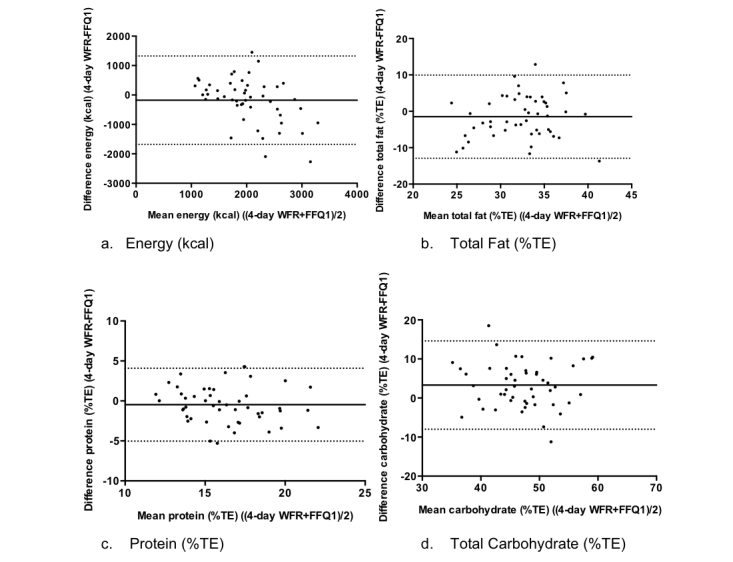
Validation study Bland-Altman plots for (a) energy, (b) total fat, (c) protein, and (d) carbohydrate with the mean difference and limits of agreement. The solid line represents the mean difference and the dotted lines represent the limits of agreement.

Bland-Altman plots for mean energy (kcal), total fat (%TE), protein (%TE) and carbohydrate (%TE) for the 4-day WFR and FFQ1 are shown in Figure 4. Overall, less than 5% of cases fell outside of the limits of agreement for all plots indicating good agreement between the methods. The mean difference (bias) between energy intakes was relatively small (178 kcal/day) with greater values being estimated in the Food4Me FFQ, as was the case for energy derived from total fat and protein.

Correlation coefficients for estimates of energy and nutrient intakes and cross-classification of quartiles of mean daily intakes between 4-day WFR and FFQ1 are presented in Table 6. Correlation coefficients ranged from .23 (vitamin D) to .65 (protein, %TE) with a mean value of .47. Correlation was significant for the majority of nutrients at the *P*<.01 level, with the exception of total fat (%TE), PUFA (%TE), and vitamin D. Retinol and vitamin E showed significant correlation at the *P*<.05 level. The percentage of participants classified into quartiles of exact agreement ranged from 22% (total fat, %TE) to 53% (MUFA, g). For classifications of exact agreement plus adjacent, values were consistently high, ranging from 65% (sodium) to 88% (total fat, g, and total sugars, g, %TE). The mean percentage of participants classified into quartiles of disagreement was 16% with less than 4% of participants classified into extreme disagreement.

**Table 6:** Unadjusted correlation coefficients and cross-classification of quartiles of mean energy and nutrient intakes derived from the online Food4Me FFQ and 4-day WFR (n=49).

Nutrient^a^	Correlation	Quartiles, %			
		Exact agreement^b^	Exact agreement plus adjacent^c^	Disagreement^d^	Extreme disagreement^e^
Energy (kcal)	.53^f,h^	41	84	12	2
Total fat (g)	.56^h^	37	88	10	2
Total fat (%TE)	.27	22	76	16	8
SFA (g)	.48^h^	37	82	14	4
SFA (%TE)	.38^f,h^	24	78	14	8
MUFA (g)	.56^h^	53	86	6	8
MUFA (%TE)	.45^h^	47	86	8	6
PUFA (g)	.45^h^	49	76	22	2
PUFA (%TE)	.24	27	71	24	4
Protein (g)	.59^h^	45	84	14	4
Protein (%TE)	.65^f,h^	45	86	14	0
Carbohydrate (g)	.43^f,h^	37	82	10	8
Carbohydrate (%TE)	.59^f,h^	49	82	18	0
Total sugars (g)	.60^f,h^	41	88	10	2
Total sugars (%TE)	.61^f,h^	45	88	10	2
Alcohol (g)	.61^h^	45	80	16	4
Calcium (mg)	.47^f,h^	41	73	17	0
Total folate (µg)	.58^h^	45	86	10	4
Iron (mg)	.50^h^	41	82	14	4
Total carotene (µg)	.42^h^	33	78	18	4
Riboflavin (mg)	.50^h^	45	84	14	2
Thiamin (mg)	.60^h^	43	82	16	2
Vitamin B6 (mg)	.44^h^	37	78	20	2
Vitamin B12 (µg)	.46^h^	39	78	20	2
Vitamin C (mg)	.54^h^	37	84	14	2
Retinol (µg)	.31^g^	37	76	18	6
Vitamin D (µg)	.23	27	67	27	6
Vitamin E (mg)	.30^g^	33	78	14	8
Sodium (mg)	.37^h^	49	65	31	4
Salt (g)	.37^h^	49	65	31	4

^a^MUFA: monounsaturated fatty acids; PUFA: polyunsaturated fatty acids; RE: retinol equivalents; SFA: saturated fatty acids; TE: total energy.

^b^Exact agreement, % of cases cross-classified into the same quartile.

^c^Exact agreement plus adjacent, % of cases cross-classified into the same or adjacent quartile.

^d^Disagreement, % of cases cross-classified 2 quartiles apart.

^e^Extreme disagreement, % of cases cross-classified into extreme quartiles.

^f^Pearson correlation.

^g^ *P*<.05.

^h^ *P*<.01.

#### Comparison of Food Group Intakes Between the Food4Me FFQ and 4-Day WFR

To assess differences in food group intakes between 4-day WFR and FFQ1, food items were categorized into 35 food groups. Correlation coefficients and cross-classification of mean food group intakes are presented in Table 7. SCC ranged widely from .11 (soups, sauces and miscellaneous foods) to .73 (yogurts) with a mean value of .2. Correlations were significant for the 63% of food groups (22 of 35).

The percentage of participants classified into quartiles of exact agreement ranged from 18% (nuts and seeds, herbs and spices) to 55% (teas and coffees). For classifications of exact agreement plus adjacent, values were high ranging from 55% (soups, sauces and miscellaneous foods) to 90% (milk, chipped, fried and roast potatoes, teas and coffees, and other beverages) with a mean of 78%. The mean percentage of participants classified into quartiles of disagreement was 17% and for extreme disagreement was 5%.

**Table 7:** Spearman correlation coefficients (SCC) and cross-classification of quartiles of food group intake derived from the online Food4Me FFQ and 4-day WFR (n=49).

Food group	SCC	Quartiles, %			
		Exact agreement^a^	Exact agreement plus adjacent^b^	Disagreement^c^	Extreme disagreement^d^
Rice, pasta, grains and starches	.34^e^	45	73	22	4
Savories (lasagne, pizza)	.16	22	76	22	2
White bread (rolls, tortillas, crackers)	.46^f^	42	73	22	4
Wholemeal and brown breads and rolls	.33^e^	31	65	29	6
Breakfast cereals and porridge	.27	31	78	14	8
Biscuits	.47^f^	41	78	16	6
Cakes, pastries and buns	.31^e^	31	73	18	8
Milk	.68^f^	51	90	8	2
Cheeses	.46^f^	41	82	14	4
Yogurts	.73^f^	43	96	4	0
Ice cream, creams and desserts	.21	35	78	18	4
Eggs and egg dishes	.55^f^	43	76	20	4
Fats and oils (eg, butter, low-fat spreads, hard cooking fats)	.35^e^	35	80	12	8
Potatoes and potato dishes	.38^f^	31	71	20	8
Chipped, fried and roasted potatoes	.52^f^	39	90	8	2
Peas, beans and lentils and vegetable and pulse dishes	.23	24	76	16	8
Green vegetables	.44^f^	37	86	10	4
Carrots	.14	20	82	16	2
Salad vegetables (eg, lettuce)	.23	39	69	18	12
Other vegetables (eg, onions)	.15	41	73	10	16
Tinned fruit or vegetables	.16	29	71	22	6
Bananas	.45^f^	29	80	20	0
Other fruits (eg, apples, pears, oranges)	.47^f^	49	76	22	2
Nuts and seeds, herbs and spices	.23	18	78	14	8
Fish and fish products/dishes	.60^f^	49	82	16	2
Bacon and ham	.53^f^	39	84	16	0
Red meat (eg, beef, veal, lamb, pork)	.26	43	80	16	4
Poultry (chicken and turkey)	.58^f^	45	86	10	4
Meat products (eg, burgers, sausages, pies, processed meats)	.20	27	78	22	0
Alcoholic beverages	.59^f^	43	73	22	4
Sugars, syrups, preserves and sweeteners	.36^f^	37	76	16	8
Confectionary and savory snacks	.25	29	71	22	6
Soups, sauces and miscellaneous foods	.11	24	55	31	14
Teas and coffees	.62^f^	55	90	6	4
Other beverages (eg, fruit juices, carbonated beverages, squash)	.66^f^	43	90	8	2

^a^Exact agreement, % of case cross-classified into the same quartile.

^b^Exact agreement plus adjacent, % of cases cross-classified into the same or adjacent quartile.

^c^Disagreement, % of cases cross-classified 2 quartiles apart.

^d^Extreme disagreement, % of cases cross-classified into extreme quartiles.

^e^ *P*<.05.

^f^ *P*<.01.

### Usability Rating

Mean values and standard deviations for responses to the dietary record usability-rating questionnaire are shown in Table 8.

The Food4Me FFQ was considered significantly easier and less time consuming to complete than the 4-day WFR. However, the 4-day WFR was rated as significantly more interesting than the Food4Me FFQ, making participants reflect more on their food intake. Participants were more willing to complete further Food4Me FFQ than 4-day WFR.

**Table 8:** Responses to Dietary Record Usability-Rating Questionnaire (n=48).

Question	Questionnaire^a^, mean (SD)		*P* value^b^
	Food4Me FFQ	4-day WFR	
1. Easy to complete	1.89 (0.71)	2.13 (0.88)	<.001
2. Too time consuming	3.43 (1.09)	3.00 (0.94)	<.001
3. Interesting to complete	2.20 (0.69)	2.07 (0.72)	.006
4. Made me reflect on my intake	2.13 (0.58)	1.89 (0.77)	.002
5. I would be willing to complete more	1.78 (0.70)	2.07 (0.77)	<.001

^a^1=strongly agree, 2=agree, 3=neither agree nor disagree, 4=disagree, 5=strongly disagree.

^b^ *P* value derived using 2-samples paired *t* test.

## Discussion

### Main Findings and Comparisons With Other Work

Previous validation of the Food4Me FFQ has demonstrated good agreement with the printed EPIC-Norfolk FFQ for the estimation of food and nutrient intake. In this study, participants were asked to complete the Food4Me and EPIC-Norfolk FFQ in a random order, 4 weeks apart. Good agreement between cross-classifications of daily energy and nutrient intakes, estimated using the 2 FFQ, demonstrated the utility of the Food4Me FFQ for ranking individuals based on their nutrient intake. However, it was noted that further testing of the Food4Me FFQ was required to establish its wider utility [18]. The present study thus aimed to demonstrate the reproducibility of the Food4Me FFQ and its validity against a 4-day WFR.

Overall, the Food4Me FFQ demonstrated good reproducibility for the estimation of intakes of nutrients and food groups. Reported energy intakes were significantly lower with the second administration of the Food4Me FFQ, but correlations between energy intakes were high (*r*=.77). Correlation coefficients for nutrient intakes ranged from .65-.90, showing above-average performance compared with the range of .50-.80 proposed by Willet [15]. The mean unadjusted correlation coefficient (*r*=.75) for energy and nutrient intake compared well with previous studies on both computerized [21,30-31] and non-Web-based FFQ [23,32-35]. Associations between food group intakes were similarly strong with an average unadjusted SCC of .75; previous studies have reported correlations of .66 and .72 [36-37]. Cross-classification analysis of repeated measures of intakes of energy, nutrients, and food groups indicated a high level of reproducibility with classification into quartiles of exact agreement plus adjacent averaging 92% for energy and nutrient intake and for food group intakes. Cross-classifications were within the range reported by previous studies [21,32,38]. Bland-Altman plots demonstrated a good level of reproducibility for energy-controlled total fat, protein, and carbohydrate intake, which reinforces evidence for the reliability of the Food4Me FFQ.

Estimated energy and nutrient intakes were higher on the first administration of the Food4Me FFQ than on the second administration. This pattern has been observed in numerous other reproducibility studies [21,31-32], and is proposed to result from learning effects and questionnaire fatigue [39]. The above-average reproducibility of the Food4Me FFQ could be attributed to the addition of food photographs to the FFQ for the estimation of portion size intake. Use of tools that allow participants to report their own portion sizes tend to report higher correlation coefficients between repeat administrations [19]. The relatively short interval between repeat administrations of the FFQ is another factor likely to have contributed to the questionnaire’s good performance. Tsubono et al [40] found that correlation coefficients tended to be lower when FFQ were repeated after a long time interval (6 months to 1 year) compared with a shorter time interval (1 to 6 months), and proposed that the temporal difference may be due to changes in dietary habits which are more likely to occur with longer time intervals. In addition, it has also been suggested that for very short time intervals between administrations, respondents may remember and replicate their entries rather than reporting their diet intake accurately [23]. However, with a large FFQ containing 157 food items, as used in the present study, it is unlikely that many participants would be able to remember their earlier responses.

The degree of underreporting between the Food4Me FFQ and 4-day WFR varied, with 12 (24%) and 15 (15%) participants deemed to be underreporting in the 4-day WFR and Food4Me FFQ, respectively. Given that the WFR is described as the gold standard for assessing intake, our observation that estimates of energy intake were similar between the Food4Me FFQ and the 4-day WFR and that a smaller proportion of participants appeared to underreport with the Food4Me FFQ suggests that the Food4Me FFQ is a promising tool for estimating habitual food intake.

Overall, the results of the validation study showed moderate agreement between the Food4Me FFQ and 4-day WFR for the estimation of energy and nutrient intake. Ranks of energy and nutrient intake estimated using the Food4Me FFQ were highly comparable to the 4-day WFR with the percentage of individuals classified into quartiles of exact agreement and exact agreement plus adjacent averaging 40% and 80%, respectively. Previous studies comparing FFQ with food records have reported average exact agreement classifications between 34% and 49% [32,38,41] and exact agreement plus adjacent quartile classifications of 77% [21]. Estimates of intake showing disagreement between measurement tools in the present study were small and were comparable with the aforementioned studies. Cross-classifications of estimates of food group intake were similar to that of the nutrients, with classification into quartiles of exact agreement plus adjacent averaging 78% and Bland-Altman plots demonstrated good agreement between the 2 methods for estimates of energy and energy-adjusted macronutrient intakes.

In the present study, 22 of 30 nutrients assessed had a correlation coefficient greater than the .4 threshold that was proposed by Cade at al. [23], and 13 of 30 achieved a correlation greater than or equal to the “desirable” .5 proposed by Masson et al [42]. The average unadjusted correlation coefficient of .47 compared favorably with the range reported by similar validation studies comparing FFQ with food records: .34-.46 [31-32,34,41]. SCC for food group intakes were highly variable, ranging from .11 (soups, sauces and miscellaneous foods) to .73 (yogurts), with a mean value of .2. Similar studies have reported correlations ranging from .09 to .83 [37], .17 to .95 [36], and .09 to .58 [41] with mean values of .38, .63, and .58, respectively. However, it is difficult to compare results from these studies because the type of food records and time intervals between dietary assessments differed substantially and there may be substantial differences in the food items included in particular food groups in each of the studies. Variation between Food4Me FFQ and 4-day WFR estimates were greatest for soups, sauces and spreads, carrots, other fruit and vegetables, and tinned fruit and vegetables. It is possible that intakes of these foods might have been overestimated in the Food4Me FFQ, as has been observed previously when several food items within a food group are listed separately in a questionnaire (eg, carrots could be counted under both fresh and tinned carrots and under tinned vegetables) [43]. In addition, foods perceived as healthy, such as fruit and vegetables, are prone to overestimation in FFQ. Furthermore, because they refer to just 4 days’ intake, WFR provide a limited snapshot of an individual’s diet only and are less able to assess patterns of dietary intake than the Food4Me FFQ, which attempts to capture intakes over the previous month. It is thought that individuals may be able to more accurately estimate the consumption of some foods (eg, alcoholic beverages) than others, as was the case in the present study [21,44]. Alcohol is often considered a confounder in nutrition research given that it constitutes the difference between food and total dietary energy intake; therefore, it is important that it is estimated reliably using the Food4Me FFQ. It is also encouraging that estimates of fish products were well correlated, as these foods are eaten less frequently and may be prone to underrepresentation in 4-day WFR. However, it is surprising that some more commonly consumed foods such as breakfast cereals and porridge show much weaker correlation (*r*=.27).

Our observation that participants in the present study reported that the Food4Me FFQ was easier to use and less time consuming compared with the 4-day WFR, is promising given the movement of health service delivery toward Web-based interventions. Moreover, completion of the Food4Me FFQ was associated with less reflection by participants on their dietary intake, which is known to influence eating behavior. Minimizing the impact of a questionnaire on dietary behavior is beneficial in nutrition intervention studies to ensure that study outcomes are not biased by the methods used for dietary assessment.

### Strengths and Limitations

Strengths of the present study include the comparison of the Food4Me FFQ with the gold standard, a WFR, and the use of multiple methods to assess the validation and reproducibility of the Food4Me FFQ. In addition, this validation study had an adequate sample size [15] similar to those used in previous studies [30,45-46]. It should be noted that the validation of the Food4Me FFQ was assessed in a convenient rather than a nationally representative sample of the population, although the inclusion/exclusion criteria used were the same as those intended for the Food4Me study [16]. The use of a convenient university population, with a potentially higher education level, may have implications on the ability of the wider population to complete the online Food4Me FFQ.

Limitations of the study include the use of those recruited first to complete the 4-day WFR because these individuals may have been more motivated to comply with the guidelines. A further limitation is the use of nonconsecutive days in the 4-day WFR, which may have resulted in participants making up food intake on the days they do not record (eg, eating healthy for the record days and overconsuming between record days). However, nonconsecutive recording does have the advantage of capturing a greater diversity of food intake over a week’s period.

A potential criticism of the assessment of reproducibility is the short duration between repeated measures. It has been suggested that for very short time intervals, respondents may remember and replicate their entries rather than accurately reporting their dietary intake [23], but this is unlikely to be a significant problem in the present study where the Food4Me FFQ contained 157 food items. Remembering their responses to such a long list of questions after a period of 4 weeks is a memory challenge beyond most people’s abilities. The average reported correlation coefficient for crude total fat intake using FFQs repeated after 1 month or less was .68 [19], which compares very favorably with the correlation coefficient of .81 in the present study, showing above-average performance for the Food4Me FFQ. Cade et al [23] suggested that the time interval between repeated measures using a dietary instrument should be chosen to minimize changes in dietary intake and our use of 4 weeks fits that criterion.

### Conclusions

In conclusion, the self-administered online Food4Me FFQ demonstrates good reproducibility for the estimation of energy, nutrient, and food group intakes and moderate agreement for the assessment of energy and nutrient intakes when compared with a 4-day WFR in an adult population. Consequently, the online Food4Me FFQ was considered suitable for the assessment of dietary intake in healthy adults.

## Multimedia Appendix 1

Food4Me Food Frequency Questionnaire screenshots.

## Abbreviations

BMI: body mass index
EPIC: European Prospective Investigation of Cancer
FFQ: food frequency questionnaire
MUFA: monounsaturated fatty acids
PUFA: polyunsaturated fatty acids
RE: retinol equivalents
SFA: saturated fatty acids
TE: total energy
WFR: weighed food record
