# Supplementary material for: Online Dietary Intake Estimation: Reproducibility and Validity of the Food4Me Food Frequency Questionnaire Against a 4-Day Weighed Food Record
Source: J Med Internet Res. 2014 Aug 11;16(8):e190. doi: 10.2196/jmir.3355 (PMC4147714; doi:10.2196/jmir.3355)
Supplement: Supplementary file 1 [file jmir_v16i8e190_app1.pdf]

Food4Me FFQ Food Categories

Extranet | Food4Me

Edit Details | Log Out

Home Instructions Info Physical Activity FAQ About

Food Frequency Questionnaire (FFQ)

Please click on each of the items listed below and then answer each question. To read the instructions again, click here (opens in a new window)

- Cereal
- Bread and Savoury Biscuits
- Potatoes, Rice and Pasta
- Meat and Fish
- Dairy Products
- Fats and Spreads
- Sweets and Snacks
- Soups, Sauces, and Spreads
- Drinks
- Fruit
- Vegetables
- Dietary Habits

Save & Exit | Submit FFQ

Selection of frequency of consumption

Food Frequency Questionnaire (FFQ)

Please click on each of the items listed below and then answer each question. To read the instructions again, click here (opens in a new window)

Cereal

Bread and Savoury Biscuits

Potatoes, Rice and Pasta

How often would you have consumed each of the following in the past month?

	Portion size	Never (<1 per month)	1-3 per month	Once a week	2-4 per week	5-6 per week	Once a day	2-3 per day	4-5 per day	6+ per day
Potatoes - mashed, instant, roast										
Potatoes - boiled, jacket										
Potato dishes e.g. salads, dauphinoise										
Chips										
White rice										
Brown rice, buckwheat and barley groats										
White pasta, noodles and other grains e.g. cous cous, polenta										
Wholemeal pasta										
Lasagne, moussaka, ravioli and tortelini, filled dumplings										
Pizza, calzone										
Springrolls										
Potato or Plain Dumplings										
	Portion size	Never (<1 per month)	1-3 per month	Once a week	2-4 per week	5-6 per week	Once a day	2-3 per day	4-5 per day	6+ per day

Meat and Fish

Dairy Products

Fats and Spreads

Sweets and Snacks

Soups, Sauces, and Spreads

Drinks

Fruit

Vegetables

Dietary Habits

Save & Exit | Submit FFQ

Selection of portion sizes

Extranet Food4Me food4me+control3L@ucd.ie

Pizza, Calzone

Choose your usual portion size for this food group

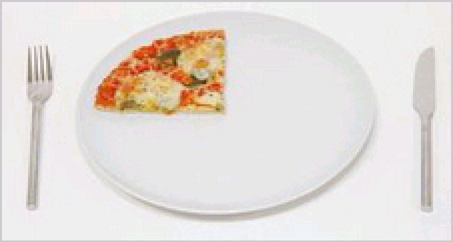
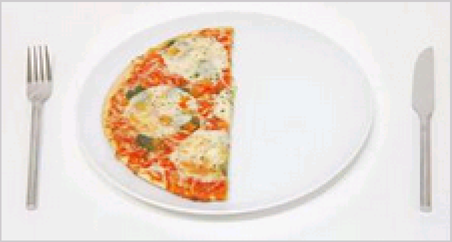
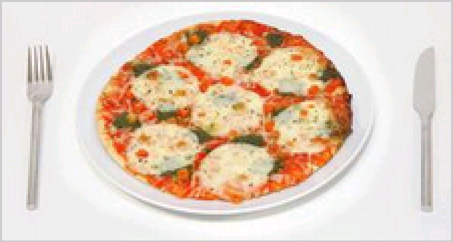

○ Very Small	○ Small	○ Small / Medium	○ Medium	○ Medium / Large	○ Large	○ Very Large

Meat and Fish

Additional dietary habits section

Dietary Habits

In the past month, were there any OTHER foods which you ate more than once a week?
Please also indicate your usual portion size.

Name of food	Number times eaten each week	Usual serving size

In the past month, how often did you add salt to food while cooking?

In the past month, how often did you add salt to food at the table?

In the past month, did you regularly use a salt substitute (e.g.: LoSalt)? If yes, what brand?

In the past month, how often did you eat fried food?

In the past month, what did you do with the visible fat on your meat?

During the past month, on average, how many times per week did you eat the following types of foods?
Please also indicate your usual portion size.

Type of food	Times per week	Usual portion size
Vegetables (not including potatoes)		
Salads		
Fruit and fruit products (not including fruit juice)		
Fish and fish products		
Meat, meat products and meat dishes (including bacon, ham and chicken)		

Supplement use

In the past 6 months, have you taken any vitamin or mineral supplements?

	Full name<br>(e.g. Centrum Advanced Multivitamin, Seven Seas Cod Liver Oil, Tesco Folic Acid 400 ug)	Amount per occasion<br>(e.g. 1 tablet ,<br>2 capsules,<br>1 teaspoon (5ml),<br>2 teaspoon (10ml),<br>3 teaspoons (15ml)	How often
Example:	*Centrum Select 50+*	*2*<br>*tablet(s)*	*Once per day*

+
